# Bionic Design of the Bumper Beam Inspired by the Bending and Energy Absorption Characteristics of Bamboo

**DOI:** 10.1155/2018/8062321

**Published:** 2018-12-02

**Authors:** Meng Zou, Jiafeng Song, Shucai Xu, Shengfu Liu, Zhiyong Chang

**Affiliations:** ^1^Key Laboratory for Bionics Engineering of Education Ministry, Changchun 130022, China; ^2^State Key Lab of Automotive Safety and Energy, Beijing 100084, China

## Abstract

This study conducted quasistatic three-point bending tests to investigate the effect of bamboo node on the energy absorption, bending, and deformation characteristics of bamboo. Results showed that the node had a reinforcing effect on the energy absorption and bending strength of the bamboo culm subjected to bending load. The experimental results demonstrated that nodal samples (NS) significantly outperform internodal samples without node (INS). Under the three-point bending load, the main failure mode of bamboo is the fracture failure. The node also showed split and fracture prevention function obviously. Based on that, a series of bionic bumper beams were designed inspired by the bamboo node. The FEM results indicated that the performance of bionic bumpers was better than that of a normal bumper with regard to bending strength, energy absorption, and being lightweight. In particular, the bionic bumper beam has the best performance with regard to bending, energy absorption, and being lightweight compared with the normal bumper under pole impact. The characteristic of the bionic bumper beam is higher than that of the normal bumper beam by 12.3% for bending strength, 36.9% for EA, and 31.4% for SEA; moreover, there was a mass reduction of 4.9%, which still needs further optimization.

## 1. Introduction

Structures in nature have evolved to fit their environment after millions of years of evolution. Bamboo is a kind of biological structure reinforced by nodes [1]. In terms of morphology and mechanics, bamboo has the flexibility to adapt to bending damage caused by natural forces and external load, such as wind, snow, and rain. The hollow structure and nodes play an important role in allowing the plant to achieve optimal stiffness and stability with the least material [2]. Moreover, the bamboo node is crucial to improve stiffness and stability of the slender bamboo culm during growth [3].

Previous researchers have discussed that these nodes have a mechanical function, improving the stiffness and strength of the culm. For example, Shao et al. [4–6] proposed that bamboo nodes were crucial to improve stiffness and stability of the slender bamboo culm during growth. Barati and Alizadeh [7] claimed that nodes support the culm to prevent failure due to local buckling. Meng et al. [8] studied the energy absorption characteristics and material parameters of the bamboo species by drop-weight and dynamic tensile tests. The results show that the energy absorption of nodal samples is greater than that of the internode samples.

For applications that require large energy absorption and bending strength, bumper beams are one of the key structures in passenger cars, which should be carefully designed and manufactured in order to achieve good impact behavior. Nowadays, some road components like roadside safety structures and vehicle safety components have been widely used [9–11], where these structures are submitted to impact loading conditions, such as the bumper and crush box. As for the bumper, there has been much research. Hosseinzadeh et al. [12] studied a commercial front bumper beam made of glass mat thermoplastic (GMT) by impact modeling using LS-DYNA, and the result shows that this kind of bumper has the advantages of being lightweight, ease of manufacturing, and reduction of production cost. Belingardi et al. [13] compare the E-Glass/epoxy pultruded bumper beam with steel and E-Glass/epoxy fabric composite beam. And the result shows that the pultruded bumper beam has comparable energy absorption capability with respect to the steel normal production solution.

The design of a crashworthy bumper beam under impact load is a very challenging job because it involves geometric nonlinear, elastic-plastic deformation and nonlinear contact deformation. Creatures in nature have experienced a long process of evolution to adapt to the environment, so they tend to have a better design that allows them to adapt to a variety of conditions: their structure often has superior mechanical and multifunctional properties. Bionics of biotical creativity engineering is the application of biological methods, which is aimed at the study and design of engineering applications; it has been successfully applied in material and structure design [14]. Yu et al. [15, 16] investigated the structure and mechanical characteristics of cattle horns, then proposed that the structure feature can also be used in the crashworthiness field. Zhao et al. [17] investigated the giant water lily leaf ribs and cactus stem for their optimal framework and superior performance. Then they extracted their structural characteristics and used them in the bioinspired design of the Lin MC6000 gantry machining center crossbeam, which has better load-carrying capacity than conventional distribution. As for bamboo, Ma et al. [18] designed a bionic cylindrical structure to mimic the gradient distribution of vascular bundles and parenchyma cells. After finite-element analysis, the results show that the load-bearing capacity of a bionic shell increased by 124.8%. Inspired by the way in which the bamboo nodes and nodal diaphragms enhance the transverse strength of bamboo, Liu et al. [19] designed the nonconvex multicorner thin-walled column by adding bulkheads in the column, and it was found that the bioinspired structure outperformed the conventional structure.

In this paper, bamboo samples with node and without node were studied to determine the influence of the node on the bending strength and energy absorption of bamboo. Meanwhile, the structure of bamboo nodes was investigated to study the influence of vascular bundles at the nodes on mechanical properties of the culm. Inspired by the structure of bamboo nodes and nodal diaphragms, a new excellent bumper beam named as the bionic bumper beam is proposed in this article, which is a high-strength, lightweight bumper beam.

## 2. Materials and Experiment

### 2.1. Materials

**Figure 1:**
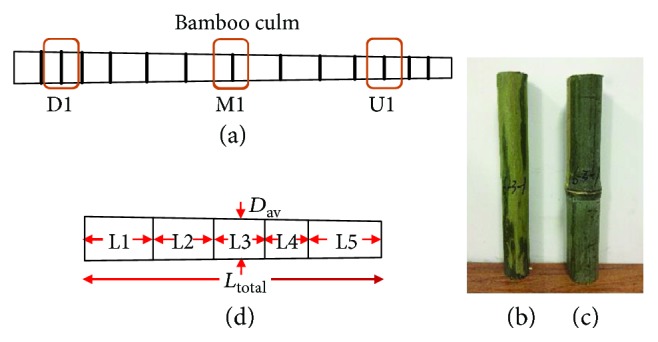
(a) Sample position; (b) internodal sample without node (INS); (c) nodal sample (NS); (d) dimension of nodal samples.

Bamboo (*Phyllostachys pubescens*) samples were collected from Jiangxi Province, near the middle Yuan River, which has subtropical monsoon humid weather. The bamboo was cut into three parts (upper, middle, and lower). When preparing samples with nodes, the nodes were positioned at the center of the samples. Samples with and without nodes were taken from neighboring sections of the same culm, thus giving an almost uniform thickness of samples, as shown in Figure 1(a). These samples were divided into internodal samples without node (named INS) and nodal samples (named NS), as shown in Figures 1(b) and 1(c). The bamboo samples were 3 years old and close to a complete plant with a culm of 20–70 mm in diameter. The wall thickness of the bamboo is about 7–30 mm. The bamboo samples were numbered and sealed before being carried back, then the bamboo samples were processed under the test requirements.

The dimension and number of bamboo samples are shown in Figure 1(d) and Table 1. Fifteen different samples located at different parts of the bamboo culm and with a different number of nodes were investigated. The samples were named by letters and numbers. The first letter “U” stands for the upper part of the bamboo, “M” for the middle part of the bamboo, and “D” for the lower part of the bamboo; the last number stands for the number of bamboo nodes.

### 2.2. Experimental Procedure

The bamboo stem's diameter tapers from basal to top [20, 21]. Its top has a smaller diameter than the basal. In this paper, its cross section was assumed as a hollow cylinder according to the ISO 22157-1:2004 [22, 23]. In this experiment, quasistatic three-point bending tests were performed with the Electronic Universal Testing Machine in Jilin University, model CSS-44100 with the load capacity of 20 kN and an internal displacement transducer.

**Figure 2:**
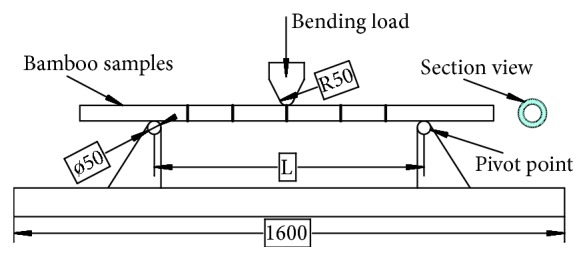
Schematic illustration of the three-point bending test setup. Note: all dimensions in millimeters.

Figure 2 shows a schematic view of the three-point bending test setup. The three-point bending test rig consists of two fixed supports and an indenter made of solid aluminum. The upper indenter was mounted to the actuator of the testing frame. The samples were placed centrally between the two fixed lower supports. A camera was mounted on the ground to obtain footage on the damage mode of samples during bending. The bamboo samples were fixed on two pivots, and the indenter compressed down with a constant bending speed of 5 mm/min. Each sample was loaded to failure, and the bending force was recorded during the bending collapse, together with the cylinder's displacement continuously, giving a force-deflection curve of the bending process.

In this paper, the bending strength (BS), radial energy absorption (EA), and radial specific energy absorption (SEA) were applied to evaluate the characteristic of the bamboo samples. The bending strength can be described by

(1) $$\begin{array}{c} BS=\sigma_{b}=\frac{3P_{\max}l}{2{bh}^{2}}, \end{array}$$

where *σ_b_* is the bending strength with a unit of MPa, *P*_max_ is the maximum broken force with the unit of N, *l* is the span distance between the two supports with the unit of mm, *h* is the sample width with the unit of mm, and *h* is the sample height with the unit of mm.

The expression of SEA [24] is shown by

(2) $$\begin{array}{c} SEA=\frac{EA}{m}, \end{array}$$

where *m* is the mass of the destroyed sample and EA [25] is the total energy absorbed by the sample, which can be obtained from

(3) $$\begin{array}{c} EA=\int_{0}^{s}F\left(s\right)ds, \end{array}$$

where *F*(*s*) is the instantaneous reacting force during bending and *s* is the bending displacement.

## 3. Mechanical Properties of Bamboo under Three-Point Bending Test

### 3.1. Structure Analysis

**Figure 3:**
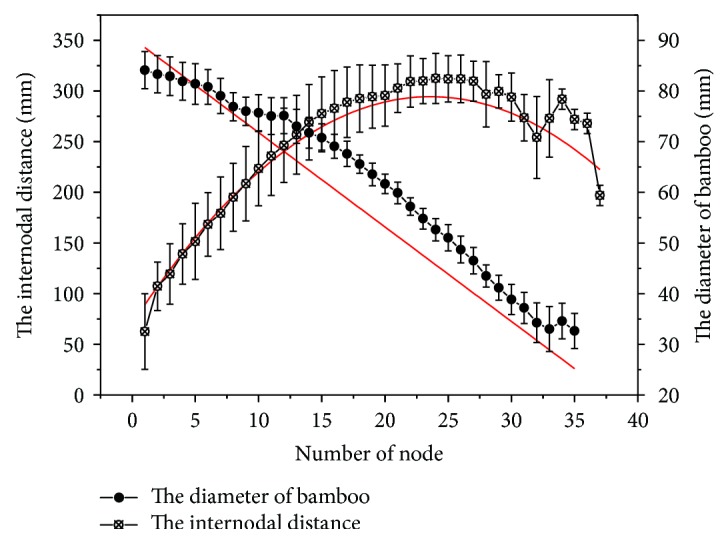
The distribution of internodal distance and diameter along the culm.

The changing rules of internodal distance and diameter along the growth direction of bamboo are shown in Figure 3, which illustrates that bamboo nodes appear at a changing interval along the stem.

According to the internodal distance along the growth direction of bamboo, the internodal distance shows a normal distribution. After nonlinear fitting regression, these data can be described by (4), which illustrates that the number of nodes at both ends is larger than that in the middle part of the bamboo.

For the bamboo's diameter, as shown in Figure 3, the diameter decreased linearly approximately along the direction of the growth, and it can also describe the mathematical model (5) by data fitting. All those facts demonstrate that bamboo is a changeable cone-shaped tubular structure.

(4) $$\begin{array}{c} y_{1}=-2836+3146^{*}e^{-2*{\left(\left(x-23.6\right)/115.4\right)}^{2}}, \end{array}$$

(5) $$\begin{array}{c} y_{2}=91-1.62^{*}x, \end{array}$$

where *x* is the number of bamboo nodes, *y*_1_ is the internodal distance, and *y*_2_ is the diameter of the bamboo.

### 3.2. Three-Point Bending Tests

For the samples with an odd number of nodes, the indenter pressed on the node, and the indenter pressed in the middle of two nodes for the samples with an even number of nodes. The inner span was adjusted so as to always have at least two nodes in the central portion of the sample.

**Figure 4:**
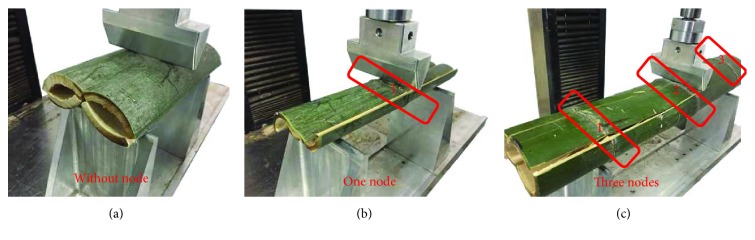
Bending test process: (a) without node, (b) one node, and (c) three nodes.

Figure 4 shows the failure mode of bamboo samples under the load of bending. When the bamboo was loaded to failure, it almost failed by splitting along its length. This is due to the fibrous structure, which makes the material much stronger in the longitudinal direction than in the transverse direction, and then split into 4 pieces. At this time, the bearing capacity reduced rapidly until compression was completed. When the load was removed after the bending test, the sample could restore a little distance, which indicated that bamboo has good elasticity and toughness. The bamboo damage was mainly brittle failure, and it almost would not produce scattered debris.

A significant global buckling mode was observed in Figure 4: a sectional crushing zone and several cracks. The localised crack subsequently led to a reduced cross-sectional area, which was associated with the decreasing load-carrying capacity after the local crack occurred at a small deflection.

### 3.3. Effect of the Existence of a Node on Bending and Energy Absorption

**Figure 5:**
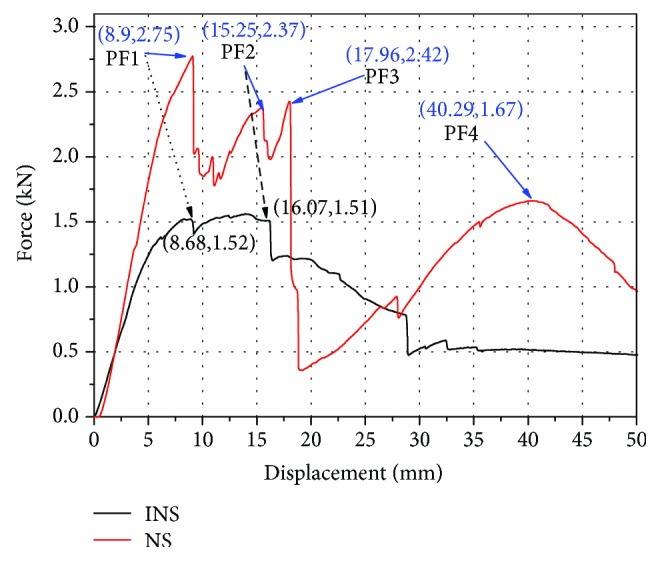
Displacement vs force of NS and INS.

**Figure 6:**
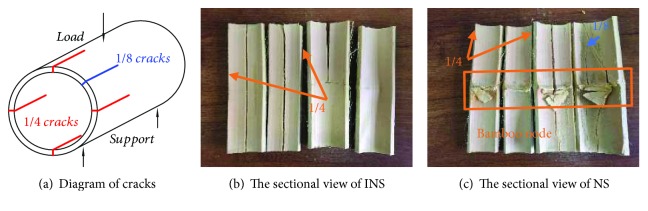
Diagram of cracks and the section view of broken samples with zero, single, and three nodes.

The experimental force-deflection curves and final broken modes under quasistatic three-point bending conditions for NS and INS are given in Figures 5 and 6. The result shows that these curves have significant differences depending on the broken modes. There are several peak points on these two curves (PF1, PF2, PF3, and PF4), and the force of the samples is linear elastic at the beginning of loading stages.

**Figure 7:**
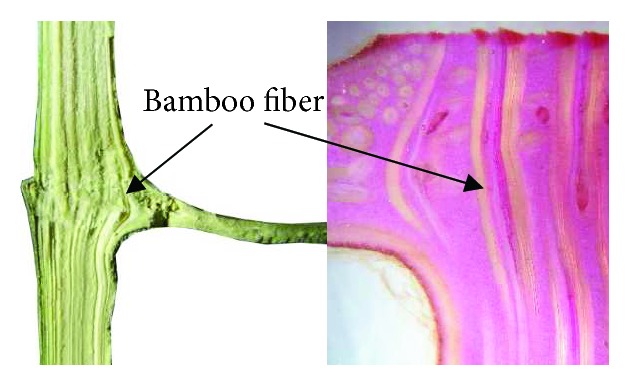
The sectional and electron macroscopic views of the internal diaphragm and external ridge of a bamboo node.

In the subsequent loading stages, when the curves reached the Peak Force1 (PF1), two cracks occurred in the direction parallel to the indenter due to the brittle fracture behavior in compression, just like the four “1/4 cracks” in Figure 6(a). The two “1/4 cracks” in Figure 6(b) propagated throughout the INS, which led to a great breakage. However, due to the existence of the node in NS, where the fibers are thick and diverge from their longitudinal orientations, as shown in Figure 7, the two “1/4 cracks” in Figure 6(c) appeared at the two pivot points firstly, leading to a slow force increase in Figure 5, and the culm of NS did not break completely.

When the curves reached the Peak Force2 (PF2), cracks of INS appeared throughout the sample at another two “1/4 cracks” of the bamboo, and the whole culm broke completely, leading to a rapid force drop. However, the bamboo wall cracked in the vicinity of the bamboo node in the NS, which also led to a rapid load drop in Figure 5.

In the subsequent loading stages, when force reached the Peak Force3 (PF3), the remaining two “1/4 cracks” also appeared on NS, and the cracks propagated throughout the sample directly due to the bamboos already being broken.

Eventually, for the last Peak Force4 (PF4), the following force of the bending is the elastic deformation stage, and the bending force started to slowly climb up to a plateau again, owing to the fact that these two kinds of bamboo samples have been divided into 4 pieces completely. Due to the fiber direction being consistent in INS, the curve went down smoothly. The bamboo fiber at the node is more and thick, which results in rising force. Then “1/8 cracks” appeared in NS, and the NS ruptured eventually.

As might be expected, more cracks along the culm, like the “1/8 cracks” in Figure 6(a), were observed in NS due to the bamboo node. Therefore, multiple cracks were formed instead of a single penetrating crack, resulting in a higher load-carrying capacity than that of the INS.

**Figure 8:**
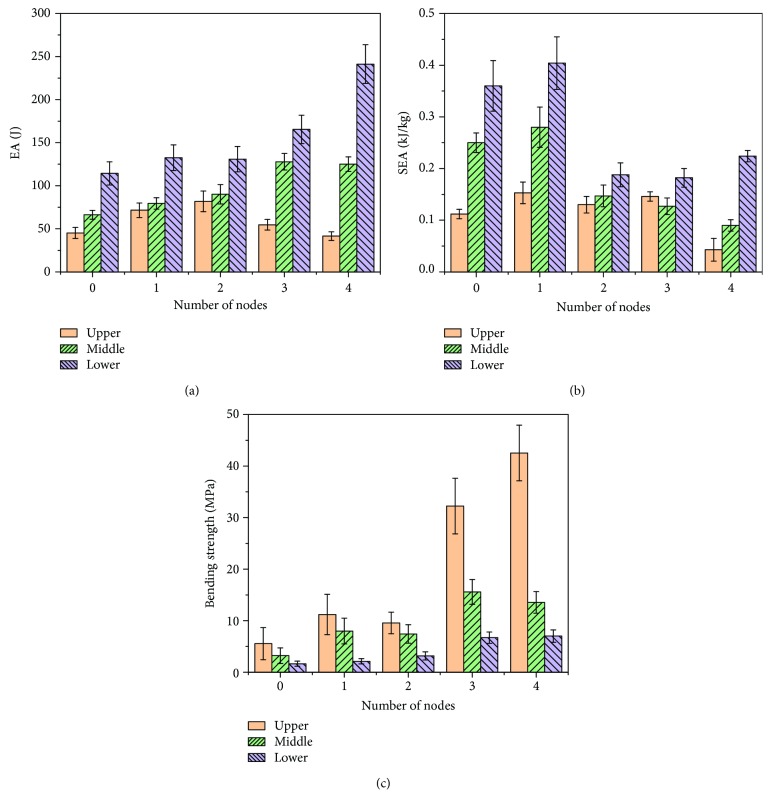
Mechanical properties of samples with a different number of nodes under bending load: EA, SEA, and BS.

Figure 5 shows that the force of the NS has four peak points (PF1/PF2/PF3/PF4), while the INS only has two peak points (PF1/PF2). The energy absorption of the sample is the integration of force during the compression process. According to Figure 8, NS (with 1 node) is 56.7% higher than INS (with 0 node) with regard to energy absorption. Moreover, NS is 31.4% higher with regard to specific energy absorption and 78.8% higher with regard to bending strength than INS. At the same time, according to the crack in Figure 6, the cracking mode and crack number of the two samples are different. INS only has 3 cracks, and the third crack didn't split entirely, while the NS has 4 cracks, which splited entirely. The additional fracture may be the result of the connection of the node, which caused the extra destruction point and then improved the bending strength and energy absorption characteristics. This suggested that the antibending ability of the node part in the intact culm was enforced by swelling of local tissues at the bamboo node.

### 3.4. Effect of the Number of Nodes on Bending and Energy Absorption

According to the above analysis, the existence of the bamboo node enhances the radial restraint force of the bamboo wall and restrains the fracture's extension, so as to improve the resistance to bending and the energy-absorbing ability.

Figure 8 shows the energy absorption and bending properties of the samples with different nodes and in different parts of the bamboo. For the upper samples, they are slender with large internodal distances. As a result, the nodes deviating off the loading point were so difficult to destroy which absorbed more energy, resulting in the bending strength being the biggest by 11.1 MPa among them. With the increase in the number of nodes, the length and mass of the samples had a greater increase and specific energy absorption decreased gradually. Because of the node's compact structure, which is close to the solid structure, the bending strength increased gradually. As for the middle samples, they have larger internodal distances and wall thicknesses. The more nodes and bigger mass caused the decline of specific energy absorption. Moreover, due to the small internodal distance, the bending strength was getting larger. In the lower part of the culm, there were small internodal distances and large wall thicknesses and mass. With the increase of the number of nodes, the specific energy absorption decreased, while the bending strength was getting larger because of the short internodal distance.

According to Figure 8, for each part of the bamboo, there was a clear tendency showing that bamboo samples with a higher number of nodes achieved a stronger stabilization of the cross section during the bending process. For example, in the case of the lower part of the bamboo, as shown in Figure 8, the bamboo node provided the culm with lateral support to force the plastic deformation to propagate towards adjacent parts. And more nodes can improve the flexural strength but lower the specific energy absorption.

The quantitative comparison between the NS and INS clearly indicates that the antibending and energy absorption ability of the bamboo sample can be significantly improved by the bamboo node. Moreover, it is evident that the number of bamboo nodes and structure size parameters significantly affect the antibending and energy absorption ability, which means that the antibending and energy absorption ability increase with the number of bamboos. For example, D4 reached nearly 3.3 and 1.8 times higher bending strength and energy absorption, respectively, than D1.

## 4. Bionic Design and Analysis

### 4.1. Bionic Bumper Beam Design

The above three-point bending test result proved that the bending and energy absorption ability of bamboo was affected by the node structure. Thin-walled metallic beams are widely applied as structural components in various engineering fields, especially in moving vehicles such as automobiles, ships, and aircrafts. Their performance under accidental impact or loading events is therefore of interest to the researchers for occupant safety considerations. In particular, the bumper beam is the primary energy absorption component in car-pole impact. In such crash scenarios, this component absorbs kinetic energy during bending collapse. At the same time, as the important components in the field of vehicle collision, the bending strength and energy absorption characteristics of the bumper beam are particularly important. At present, it is noted that in most cases, the bumper energy absorber system is mostly designed with some composite materials and normal structures for their high-energy absorption capability [12, 13, 26, 27]; however, in the energy absorber field of bionic design, there is still little research. For example, Zou et al. [28] designed a bionic tube composed of 1 bionic node and 3 bionic inner tubes with 18, 9, and 4 bionic elements in each inner tube. Numerical results indicated that the bionic design enhances the specific energy absorption of tubes.

Based on the above research on the relationship between bending and energy absorption properties and structural parameters of bamboo, the bamboo node and changeable diameter play an important role in the process of bending and energy absorption. Inspired from the way by which the bamboo nodes and nodal diaphragms enhance the transverse strength of bamboo, a series of bumper beams were proposed for improving the energy absorption property under the principle of bionic design.

**Figure 9:**
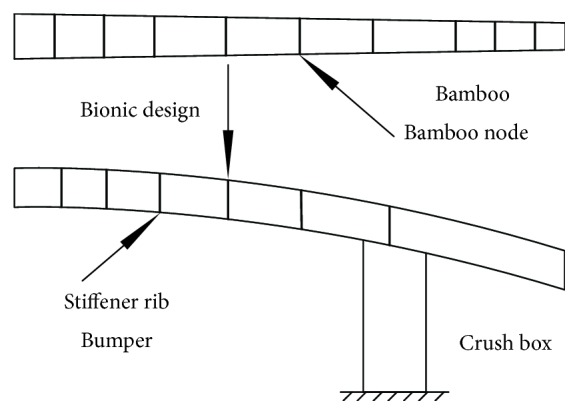
The design idea of the Bionic1 and Bionic2 bumper beams.

Figure 9 is the design idea for the bionic bumper beam, and the bionic rib is simulated by the bamboo node, which plays the role of reinforcing the strength of the bumper like the bamboo node.

**Figure 10:**
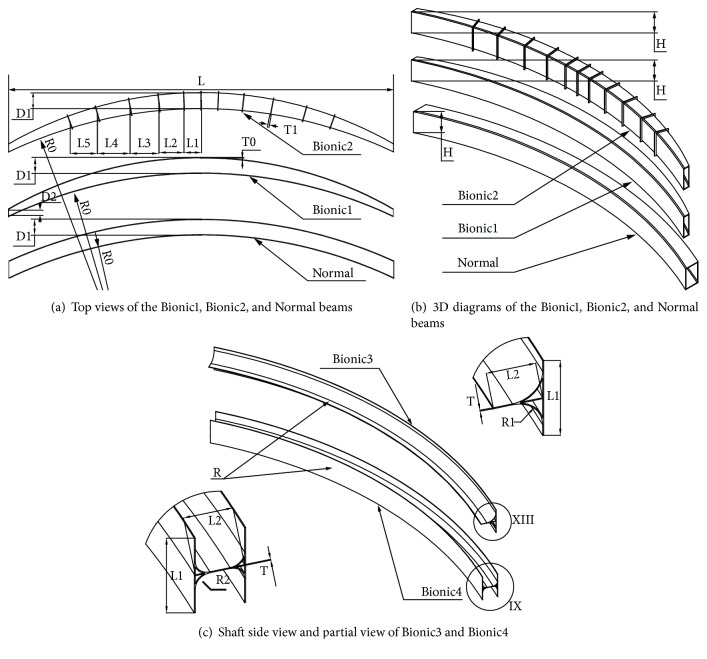
The diagram of bionic bumper beams.

For the *Bionic1* bumper beam, the beam comprised four panels. These panels located at the front/rear and top/bottom formed a beam with rectangle-shaped profiles. The distance between the front and rear panels was changing according to the changing rules of the bamboo diameter in (1). The distance is getting larger from both sides to the middle part, which gradually increases from D2 to D1, as shown in Figures 10(a) and 10(b) and Table 2.

**Figure 11:**
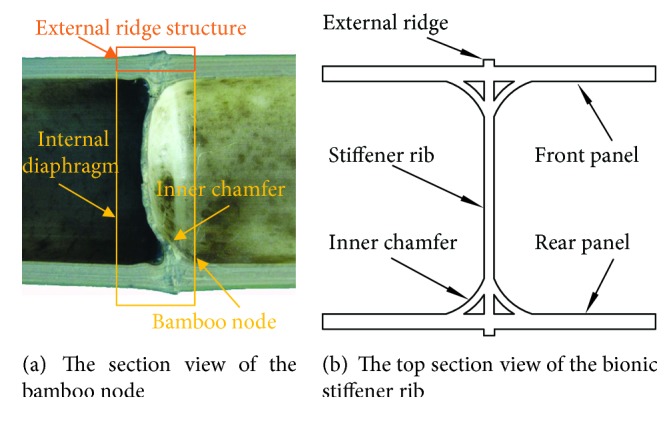
Simplified engineering design of the bionic rib according to the structure of the bamboo node.

Because of that, the cavity structure has difficulty in bearing the large load and absorbing higher energy, and the bamboo node can improve the bending performance of the bamboo. Therefore, on the basis of the *Bionic1* bumper beam, the *Bionic2* bumper beam was designed with the same cross-sectional dimension with a reinforcing *bionic rib* to solve the problem of low strength and utilization rate of the structure. These ribs were distributed like the bamboo node along the culm. The distance between two adjacent ribs was determined by the model of (2). At the same time, a *Bionic2S* was designed, in which thickness T1 of the bionic rib is thinner than that of *Bionic2*. The top view and size parameters of *Bionic1* and *Bionic2* are as shown in Figures 10(a) and 10(b) and Table 2. For the design of the *bionic rib*, as shown in Figure 11(a), the section view of the bamboo node shows that there is an internal and external ridge at the node. By engineering treatment, a simplified bionic stiffener rib was designed according to the structure of the bamboo node, which contains one stiffener rib and inner chamfer connected to the panels all around, as shown in Figure 11(b).

For comparison purposes, one kind of bumper with no rib, which is named *Normal*, was also investigated as a reference bumper beam, as shown in Figures 10(a) and 10(b) and Table 2.

Due to the great changes in mechanical properties of the bamboo which appeared at the bamboo node, the structure of the internal diaphragm plays an important role in the mechanical properties of bamboo. By engineering treatment, combined with the microscopic structure in Figure 7, two kinds of the bionic bumper were designed, the *Bionic3* and *Bionic4* bumper beams, as shown in Figure 10(c). For these two bumper beams, a design with a reinforced stiffener plate for the structure of the node was adopted to improve the strength in the bending zone. The *Bionic3* bumper beam had a single front panel and a vertical stiffener plate, while the *Bionic4* bumper beam had double panels connected by a vertical stiffener plate.

**Figure 12:**
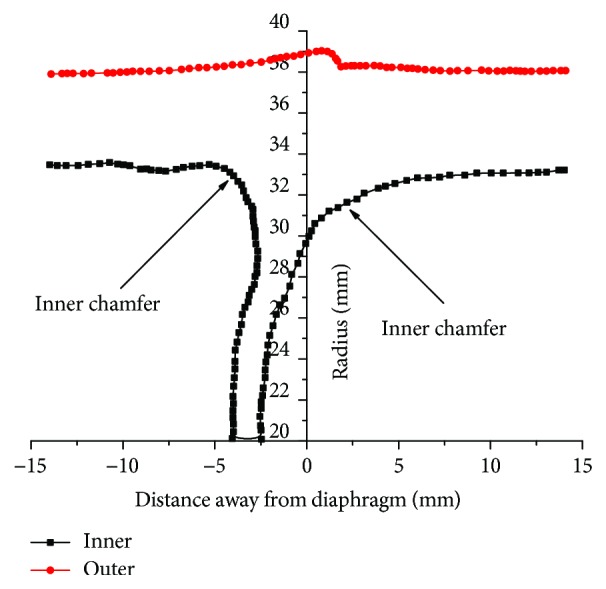
The outer and inner radii taken from a longitudinal section of the culm.

Figure 12 shows the radius size parameters of the bamboo node section view and the inner chamfer near the corner of the node. After calculation, based on the structure parameters, the range of the inner chamfer is 8–32 mm, which can guide the design of the inner chamfer in Figure 11(b) and the internal structure of R1 and R2 of the *Bionic3* and *Bionic4* bumper beams in Figure 10(b). The detailed size parameters of these bumper beams are as shown in Table 2.

### 4.2. Numerical Analysis

**Figure 13:**
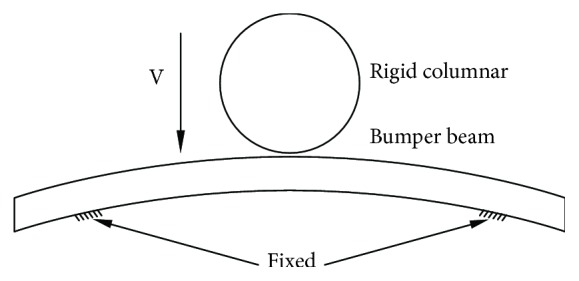
The schematic diagram of columnar collision.

At present, the automotive industry deals with a large variety of crash situations. The wide variety of accidents makes it desirable to consider them in groups with basic similarities. The largest proportion of accidents, about 60%, occurs at the front of the vehicle, and of these, pole impact is an accident situation commonly seen on roads, and they also give rise to the highest portion of deaths [29]. In this paper, these 5 kinds of bumper beams were simulated under the pole impact by finite-element software. The bumper beam was completely fixed at two points on the ground, and a rigid column with the diameter of 254 mm crashes toward the bumper beam at a certain speed. The collision diagram is as shown in Figure 13.

The FE models of bumper beams were built using the finite-element software “*Hypermesh*” and solved by LS-DYNA. The beam was modeled with the *Belytschko* thin shell elements, and the column was a rigid wall. To simulate the contact between the rigid column and beam, the “automatic surface to surface” contacts with static and dynamic friction coefficients of 0.3 and 0.2, respectively, were defined. An “automatic single surface” contact was defined for the contact of the beam itself during crushing. Stiffness-based hourglass control was employed to avoid a spurious zero-energy deformation mode, and reduced integration was used to avoid volumetric locking. The rigid column impacts the beam at the velocity of 10 m/s with the mass of 1000 kg.

The finite-element model and material parameters of the beam are as shown in Table 3. In the finite-element modeling process, the bumper beam was modeled using an elastic-plastic material constitutive model (i.e., the material model 24 in LS-DYNA). The rate-dependent effect was neglected due to the insensitivity of aluminum alloy to the relatively low strain rate.

### 4.3. Results and Discussion

**Figure 14:**
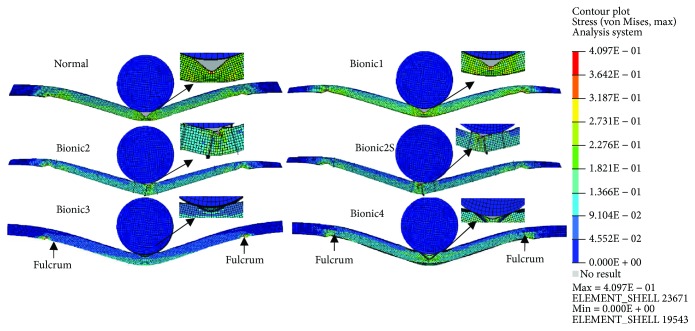
The deformation, enlarged partial view, and von Mises of the bumper's pole impact.

The deformation mode and stress pattern of 5 bumpers for pole impact are as shown in Figure 14. They have different deformation styles. For the *Normal* bumper, due to the hollow structure, a large folding with a “V” shape appeared at the middle impacting point. And the stress was mainly located at the rear flat and middle crushing point; as for the *Bionic1* bumper, the distance between the front and rear flats is short; as a result, the angle of the folding scale was large but the area was small, and the stress was mainly located at the two pivot points and middle crushing point; due to the existence of the bionic rib structure in the *Bionic2/Bionic2S* bumper beams, the strength of the beam gets stronger and the rib can make the beam more stable. The folding almost fit the rigid column at the impacting points, and the stress distributed in the whole beam uniformly.

The bumper beams of *Bionic3* and *Bionic4* are mainly made of flat and radial plates; thus, the main deformation is the laceration of the flat and radial plates. The *Bionic3* has a perfect folding, fitting the rigid column completely, which benefits from the flat front plate that is easy to fit. The rear radial plate plays an important role in the process of crushing according to the stress distribution; because the *Bionic4* has a double plate and lower rib angle, the folding was much tighter, and the stress distributed uniformly.

According to the deformation and stress distribution of the bionic and normal bumpers, the utilization of structure was low during the crushing owing to the hollow structure of *Normal* and *Bionic1* bumpers. However, the *Bionic2* bumper has the bionic rib, which made its deformation complicated and difficult. For the *Bionic3* and *Bionic4* bumper beams, the main deformation was the laceration. Therefore, the deformation and stress distribution of bionic bumpers were better than those of the normal bumper beam.

**Figure 15:**
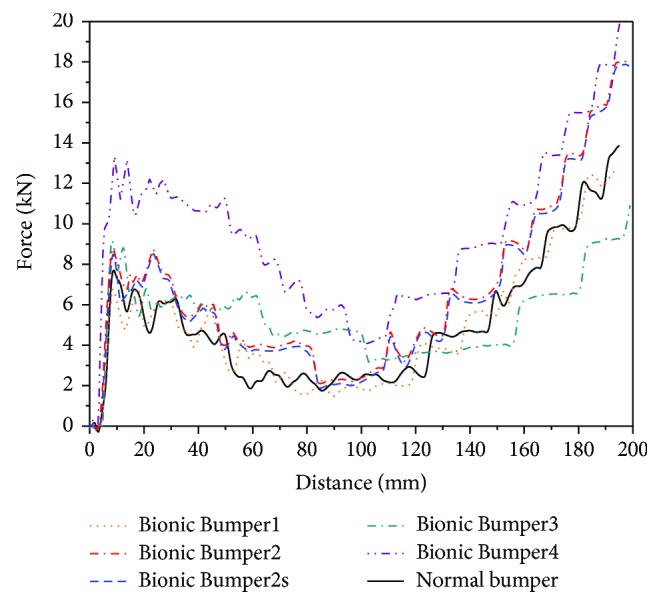
The force curve vs displacement for five bumpers.

Figure 15 shows the curves of force vs displacement for five bumpers.  Table 4 has the detailed bending and energy absorption properties of five bumpers. During the crushing distance of 200 mm, the force curves increased to the initial peak force firstly and then dropped down when the bumper was broken. Finally, the force increases again for bending and laceration.

The force curve of the *Normal* bumper is steady, which is in accordance with the deformation; *Bionic1* has the same variation range with *Normal*, and the difference between them is small, while the force of the *Bionic1* bumper is lower than that of the *Normal* bumper for the tight structure at two sides, because the structure size of *Bionic1* at the two sides is diminished, and the mass is smaller. Therefore, the SEA of the *Bionic1* bumper is higher than that of the *Normal* bumper by 7.3% but 11.3% lower than BS. For the *Bionic2*, the bionic rib made the force increase a lot, which is higher than that of the *Normal* and *Bionic1*. The characteristic of *Bionic2* is higher than that of *Normal* by 12.3% for BS, 36.9% for EA, and 31.4% for SEA. Moreover, the *Bionic2* is higher than that of *Bionic1* by 26.6% for BS, 38.3% for EA, and 22.5% for SEA, indicating that the bionic rib has a great effect on the properties of the bumper beam. As for the *Bionic2S* bumper beam, the bending strength and energy absorption are slightly lower than those of the *Bionic2* bumper beam. However, the mass of the *Bionic2S* bumper beam is lower than that of the *Bionic2* and *Normal* bumper beams, which is the lightweight effect.

The initial peak force of *Bionic3* is equal to that of *Bionic2*, but it has many large fluctuations, and the latter part of the curve tends to be jagged, indicating that the structure had unsatisfied deformation. The characteristic of *Bionic3* is higher than that of *Normal* by 11.5% for BS, 9.3% for EA, and 21.4% for SEA. However, the *Bionic4* has the highest force and initial peak force among them. The characteristic of *Bionic4* is higher than that of *Normal* by 74.6% for BS, 94.8% for EA, and 56.2% for SEA, but the mass is the biggest one among them. Above all, it can be concluded that the bionic bumper with the best performance is the *Bionic2S*, which has the 4.9% mass reduction. And the *Bionic2S* bumper beam improved by 12.3% for BS, 36.9% for EA, and 31.4% for SEA compared to the *Normal* bumper beam.

The above results indicated that the bionic method could improve the crashworthiness of a beam structure under certain conditions; however, the bionic structure still has some limitation, for example, the material is relatively simple and the structure is relatively complex. Further research and finite-element analyses should be conducted by coupling different materials or topological optimization.

## 5. Conclusions

This work has investigated the mechanical role of the nodes in the bamboo culm under bending load. Conducting three-point bending test was attempted to determine the effect of the node on culm bending strength and energy absorption. It was concluded that the result of the present work suggests that the morphological features of the node, the internal diaphragm, and the external thickening of the culm play an important role in the mechanical properties of bamboo.

The experiment result shows that the node increases the bearing area, which can be explained as an attempt to reinforce a biologically essential feature. Moreover, the microfiber of bamboo appeared thicker and to migrate. The characteristics of the samples with nodes are higher than those of the samples without nodes by 56.7% for EA, 31.4% for SEA, and 78.8% for bending strength. With the increase of the number of nodes, the bending strength is getting larger but the SEA is getting lower.

The failure mode of bamboo is mainly rupturing under the bending force. Moreover, the existence of a bamboo node can hinder the propagation of internodal cracks to make up for the defect of weak opening-mode fracture toughness along interlamination.

Based on the above investigation, a series of bionic bumpers were designed under the principle of bionic design. After simulation by the finite-element analysis, the result shows that the performance of the bionic bumper is better than that of the normal bumper with regard to bending strength, energy absorption, and being lightweight. The *Bionic2S* has the best performance. The characteristic of *Bionic2S* is higher than that of *Normal* by 12.3% for bending strength, 36.9% for EA, and 31.4% for SEA, which still need further optimization. Moreover, there was a mass reduction of 4.9% compared to the *Normal* bumper beam. The above research can provide reference and basis for crashworthiness of structure design.

## Figures and Tables

**Table 1:** Structure parameters of bamboo samples (unit: mm).

Sample	*L*_total_	*m* (g)	*D*_av_	*T*_av_	*L*_1_	*L*_2_	*L*_3_	*L*_4_	*L*_5_	Moisture content (%)
U0	250	124.61	41.91	4.65	250					20.81
U1	250	158.89	48.68	5.06	125	125				22.76
U2	735	442.46	44.41	5.27	145	440	150			16.18
U3	949	661.22	48.15	5.4	150	320	329	150		15.91
U4	1580	977.1	40.47	4.78	150	420	430	430	150	18.28
M0	247	247.17	58.54	5.65	247					25
M1	250	281.85	63.04	5.96	125	125				29.87
M2	658	787.37	63.4	6.61	145	363	150			31.47
M3	862	1088	65.48	6.3	150	277	285	150		28.12
M4	1425	1822.73	65.43	6.78	150	390	380	355	150	29.82
D0	232	337.7	74.55	7.55	232					25.88
D1	250	424.7	76.75	7.96	125	125				31.6
D2	545	861.18	71.12	7.79	145	250	150			20.78
D3	777	1312.32	72.12	8.07	145	230	252	150		18.4
D4	1052	1788.92	71.81	7.84	147	230	255	270	150	23.04

**Table 2:** Structure parameter of bionic bumpers (mm).

Parameter	Normal	Bionic1	Bionic2	Bionic2S	Bionic3	Bionic4
L	1200	1200	1200	1200	1200	1200
H	80	80	80	80	—	—
L1	—	—	52	52	80	80
L2	—	—	76	76	50	50
L3	—	—	88	88	—	—
L4	—	—	100	100	—	—
L5	—	—	85	85	—	—
R	—	—	—	—	1400	1400
R0	1400	1400	1400	1400	—	—
R1	—	—	—	—	30	—
R2	—	—	—	—	—	15
D1	50	50	50	50	—	—
D2	50	25	25	25	—	—
T	—	—	—	—	2	2
T0	2	2	2	2	2	2
T1	—	—	2	1	—	—
*m* (kg)	1.635	1.509	1.705	1.555	1.47	2.04

**Table 3:** Finite-element model parameters.

Model parameters	Parameter values
Element size	5 mm
Density of the material	2.7*e*^−6^kg/mm^3^
Elastic modulus	70 GPa
Yield strength	0.25 GPa
Poisson's ratio	0.3
Collision mass	1000 kg
Collision speed	10 m/s

**Table 4:** The impact performance parameters of the bumpers.

	BS (MPa)	EA (kJ)	SEA (kJ/kg)
Normal	50.23	0.97	0.593
Bionic1	44.53	0.96	0.636
Bionic2	56.39	1.328	0.779
Bionic2S	56.04	1.282	0.824
Bionic3	55.99	1.06	0.72
Bionic4	87.68	1.89	0.926

## Data Availability

We have submitted the raw data used in our manuscript and the other supplementary date are shown in the attachment. Other researchers can access the data supporting the conclusions of the study. (1) The nature of the data is the source data of the image in the paper; (2) the data can be accessed on the submitting system or through email to iansongjiafeng@163.com; (3) there are no restrictions on the data access.
